# Diagnosis of an Arachnoid Cyst in a Patient Presenting With Uncommon Depressive Symptoms: Case Report

**DOI:** 10.1002/ccr3.73570

**Published:** 2026-09-23

**Authors:** Ruth Arias‐Hidalgo, Felipe Ortuño, Sergio Manuel Solís‐Barquero, Maria Fernández‐Seara, Reyes García de Eulate, Aziz Yarkin

**Affiliations:** ^1^ Department of Psychiatry and Clinical Psychology Clínica Universidad de Navarra Pamplona Navarra Spain; ^2^ Instituto de Investigación de Navarra (IdisNa) Pamplona Navarra Spain; ^3^ Department of Radiology Clínica Universidad de Navarra Pamplona Navarra Spain; ^4^ University of Navarra Pamplona Navarra Spain

**Keywords:** arachnoid cysts, mental disorders, neuroimaging, neurosurgery

## Abstract

Arachnoid cysts are most commonly asymptomatic and usually do not need any active treatment. Currently, psychiatric symptoms are seldom taken into consideration during treatment evaluations in this patient population. We present this case of a young man who was treated in our hospital for signs of depression. Due to his atypical presentation, we decided to perform a brain MRI, which detected the presence of an arachnoid cyst located in the right insular region. A further diffusion tensor imaging tractography study was performed, which showed variations in the fractional anisotropy asymmetry indexes. Our case supports the notion that arachnoid cysts can induce different psychiatric conditions, even in the absence of alterations in the neurological examination. Furthermore, it supports the importance of ordering brain imaging studies to rule out the presence of arachnoid cysts, especially if the clinical manifestations and/or age of presentation are unusual. In light of the knowledge of functional neuroanatomy, there is a need for more neuroimaging studies in order to clarify the mechanisms by which certain brain‐occupying lesions can induce structural and functional changes.

## Key Clinical Message

Arachnoid cysts can present with a wide range of psychiatric symptoms, which in some cases are resistant to medical treatment and benefit from surgical intervention. Due to a lack of concrete evidence, there is controversy regarding their surgical intervention. Further research is necessary to demonstrate the benefit of surgical intervention.

**Abbreviations**
CT: computed tomography
DTI: diffusion tensor imaging
FA: fractional anisotropy
FDG: fluorodeoxyglucose
MD: mean diffusivity
MMPI: minnesota multiphasic personality inventory
MRI: Magnetic Resonance Imaging
PET: Positron Emission Tomography
RD: radial diffusivity
SCIP‐S: minnesota multiphasic personality inventory
SCIPS‐S: Screen for Cognitive Impairment in Psychiatry‐Spanish Version

## 1 Introduction

An arachnoid cyst is a collection of cerebrospinal fluid that represents 1% of space‐occupying brain lesions, with a higher prevalence in males [1]. It is considered a benign neurological tumor, and most of them are of congenital origin. They are usually an incidental MRI finding and are located most commonly in the Sylvian fissure [2]. The majority of the cysts are asymptomatic. As for the symptomatic cysts, the patients most frequently present with headaches, transient ischemic attacks, ataxia, seizures, dizziness, and visual changes [2]. In general, arachnoid cysts may be secondary to the developmental agenesis of cortical regions [3]. Due to this fact, either because of the cyst itself or because of the agenesis of cortical regions, normal structural and functional development is compromised, leading to alterations in the maturation of the personality or increased vulnerability to diverse and atypical mental disorders [4].

In this report, we present a case of a young patient manifesting atypical psychiatric symptoms alongside an arachnoid cyst located in the right temporal lobe.

## 2 Case Presentation/Examination

Our patient was a 21‐year‐old Caucasian male. He was single and the oldest of two brothers. He earned a bachelor's degree in pharmacy. The patient had come for an outpatient consultation at our hospital presenting with psychopathological alterations, characterized by symptoms of depression and anxiety of mild to moderate intensity. He had previously been diagnosed with schizoid personality disorder and a mild depressive episode at a different hospital. Regarding his current symptoms, he presented with low mood, feelings of sadness, occasional episodes of crying, loss of interest in activities, social isolation, passive ideas of death, obsessiveness, and internal restlessness with nervousness. He did not present a formal alteration of thought. His speech was scarcely informative. Our observations could be summarized as follows: we perceived a person with low vital tone and expressiveness, certain partial apathy, and anhedonia. He expressed being emotionally labile, alongside increased irritability. Furthermore, he reported occasional states of psychic anxiety with somatic symptoms such as palpitations, sweating, trembling, and passive ideas of death without active ideation. His appetite was not affected. The patient also reported new onset insomnia and difficulty in the ability to concentrate with subjective memory complaints. We did not observe any evidence of sensory and perception alterations in the experience of the ego. The patient continued to work as an employee in his family's pharmacy, although he faced difficulties due to a lack of motivation and absent‐mindedness, which he attributed to a lack of attention and memory. The patient stated that, for as long as he could remember, he had always experienced discomfort in social situations. Regarding his developmental history, according to information provided by his parents, since childhood, he had been introverted with little spontaneous communication and poor emotional expression, not very sociable, and preferred to play alone. He did not present any notable health problems during childhood. Adaptation to school was normal, except for fear and crying when being separated from his parents at the beginning of schooling. He did not have any special difficulties in academic performance, nor did he show any behavioral alterations. Regarding substance use, he acknowledged sporadic use of alcohol and cannabis in recent years.

### 2.1 Magnetic Resonance Imaging (MRI)

**FIGURE 1:**
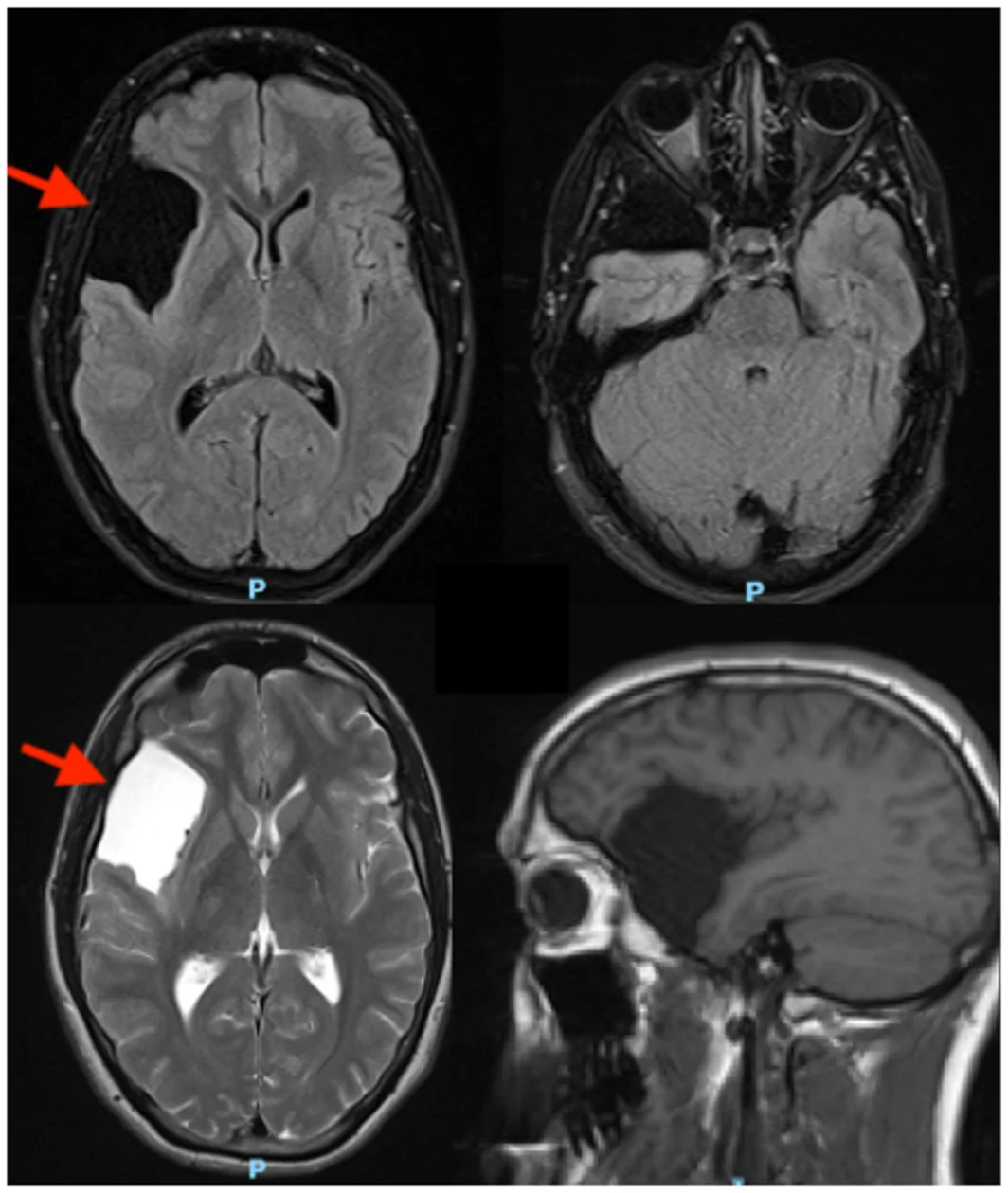
Magnetic Resonance Imaging demonstrating the arachnoid cyst located in the right temporal region.

Since this was our first time seeing this patient, we followed our hospital's protocol and performed a brain MRI. This was the patient's first episode of depression with uncommon characteristics, which further justified the use of imaging studies (according to the clinical history, his previous depressive episode was not accompanied by cognitive complaints or difficulties in social interactions). The image showed a large simple arachnoid cyst extending cranially from the right middle cranial fossa, altering the morphology of the right frontal lobe without causing mass effect (Figure 1). We believed that the cyst was probably of congenital origin.

### 2.2 Positron Emission Tomography (PET)

**FIGURE 2:**
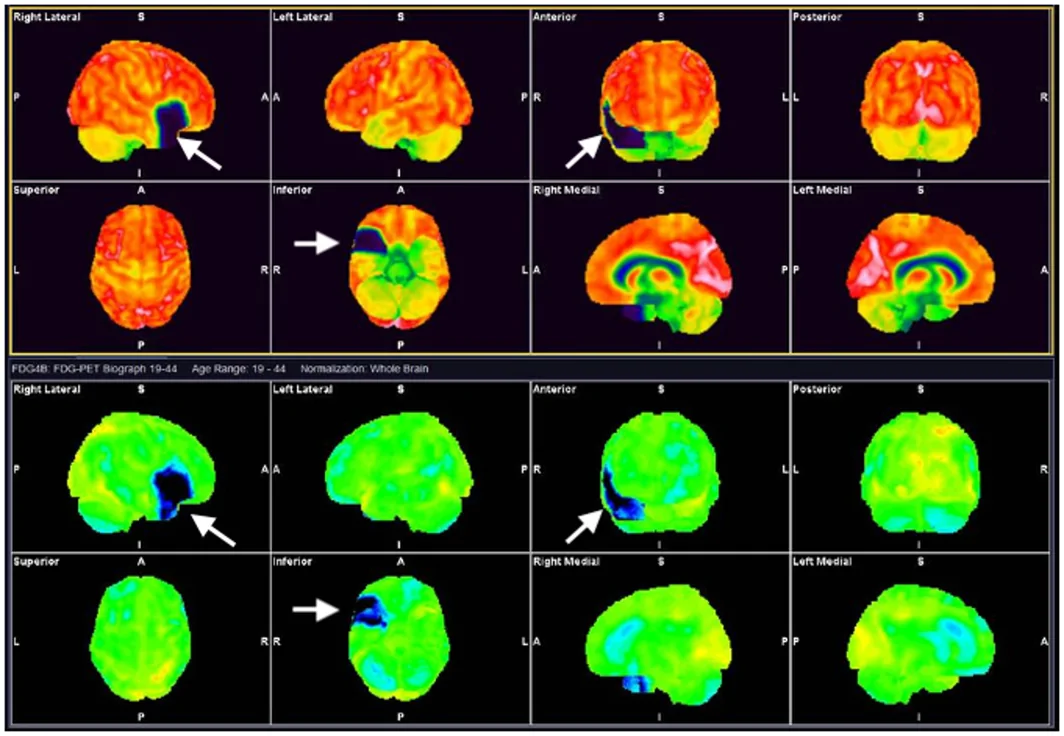
Positron Emission Tomography demonstrating hypometabolic activity in the right prefrontal cortex, insular lobe, and a part of the orbitofrontal cortex.

In order to verify the possible functional involvement of the cyst, a PET/CT scan was performed. The scan was conducted 46 min after intravenous (I.V.) injection of 320 MBq of 18 FDG. The image showed an extensive arachnoid cyst in the region of the right insula extending to the inferior temporal pole and slightly displacing adjacent cortico‐subcortical structures. Furthermore, the cyst caused ventricular dilatation. Hypometabolic activity was observed in the right prefrontal cortex, insular lobe, and in a part of the orbitofrontal cortex (Figure 2).

## 3 Methods (Differential Diagnosis, Investigations, and Treatment)

Based on the imaging findings, we decided to request an evaluation by the neurosurgery department. According to the neuroradiological findings and clinical criteria, they rejected surgical intervention for the cyst and instead recommended radiological and clinical follow‐up. We initiated treatment with sertraline 50 mg/day, and the patient was advised to follow up periodically with a local psychiatrist.

Two years into the treatment, he reported a relative improvement in mood with pharmacological treatment. Unfortunately, once again he was experiencing a low mood accompanied by loss of interest, increased irritability, and occasional thoughts of death without suicidal ideation. At this time, there were complaints of internal restlessness with occasional anxiety crises and difficulty sleeping. The intensities of depressive symptoms and baseline anxiety were moderate and mild, respectively (Hamilton Depression Rating Scale of 18 points and Hamilton Anxiety Rating Scale of 11 points). The patient especially complained of difficulty concentrating and absent‐mindedness on a more frequent basis. Furthermore, the patient experienced anxiety about social interactions, which manifested itself as avoidance and discomfort during said social interactions. His parents commented on his poor communication with family members and his tendency to be anxious and avoidant. They believed that the patient's poor communication skills were an obstacle that hindered his interaction with the clients at the pharmacy where he worked. The patient's symptomatic presentation made us consider adjustment disorder as a differential diagnosis, but we discarded this possibility due to symptoms being present before his allocation at his work in the family pharmacy.

### 3.1 Minnesota Multiphasic Personality Inventory (MMPI)

In order to assess the patient's personality and cognitive complaints, a personality test and a cognitive function test were performed. The predominant traits that stood out in the test were social introversion, depression, and psychasthenia. These findings suggested a person with a reserved character who avoided getting emotionally involved in relationships. Furthermore, he presented characteristics of insecurity, indecisiveness, doubtfulness, ambivalence, pessimism, and preoccupation with his personal deficiencies, with a limited capacity to tolerate pressures and low self‐esteem. The test helped us uncover a tendency toward perfectionism, alongside a feeling of guilt when he did not obtain the desired performance. While completing the MMPI, the patient reported difficulty concentrating and little self‐confidence.

### 3.2 Screen for Cognitive Impairment in Psychiatry (SCIP‐S)

The patient's performance in verbal fluency was below the expected level, which may imply difficulties in accessing lexical knowledge. The rest of the clinical areas explored were within the normal limits, which indicated an overall cognitive performance within the normal range.

### 3.3 Cerebral Magnetic Resonance Imaging (MRI)

**FIGURE 3:**
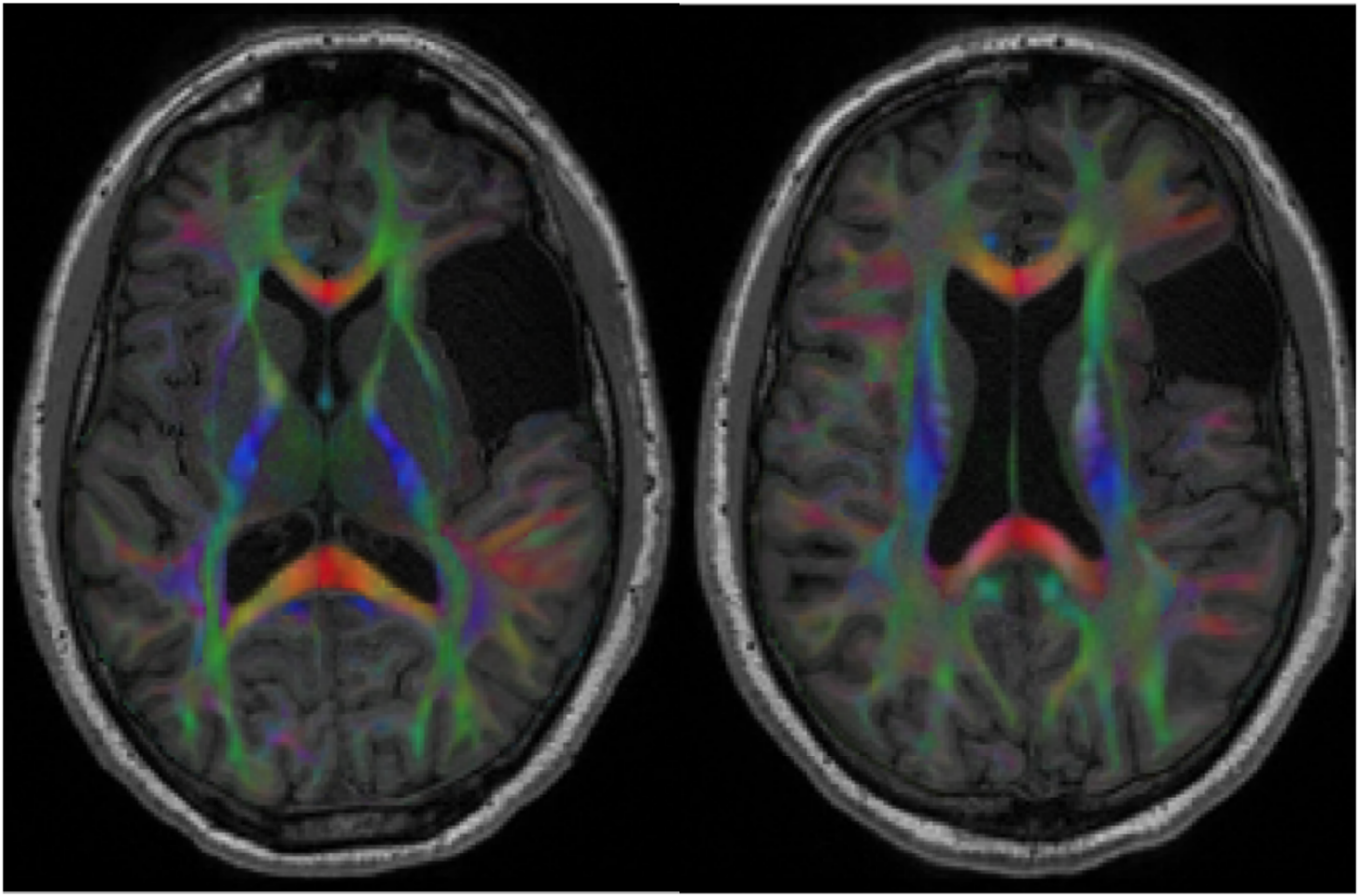
Diffusion tensor imaging demonstrating the relationship between the arachnoid cyst and the white matter structures.

In order to evaluate the evolution of the cyst and further explore the possible relationship to psychiatric symptoms, brain imaging tests were performed once more. The repeated brain MRI showed an arachnoid cyst in the right insular region without changes with respect to the previous images. A further DTI MRI (Figure 3) was performed, with an analysis of fractional anisotropy (FA), mean diffusivity (MD), and radial diffusivity (RD). The right side of most of the tracts analyzed had a higher FA value than the left side, except in the internal capsule (anterior and posterior arm), the cerebral and cerebellar peduncle, and the superior fronto‐occipital fasciculus. The major differences were in the cingulum (hippocampus), tapetum, uncinate fasciculus, and fornix. Regarding MD and RD, the values were higher for the right hemisphere (side of the lesion) than for the left hemisphere, indicating less restriction.

## 4 Conclusion and Results (Outcomes and Follow‐Up)

One year later, the patient came for his second follow‐up. In comparison with his previous follow‐up, he noted improvements regarding his depressive symptoms. Even though he still experienced anxiety during social interactions at work, he had learned to handle the anxiety, which made his time at work less stressful. His sleep disturbances had not experienced much change. He reported taking alprazolam 0.25 mg if he encountered difficulties falling asleep. Regarding his interpersonal relationships, he reported being more socially active, which had positive results in his friendships.

We believe that neuropsychiatric manifestations should be considered alongside neurological manifestations when deciding therapeutic approaches. Further investigations, such as prospective cohorts, should be conducted in order to support this notion. Furthermore, this case highlights the importance of performing brain imaging studies in patients suffering from a first episode of a psychiatric disorder, regardless of whether the episode is psychotic, affective, or cognitive in nature, especially if there are unconventional features or a poor response to treatment.

## 5 Discussion

In the literature, several articles have been published regarding the relationship between arachnoid cysts and psychiatric symptoms. Generally, the most common psychiatric symptoms that have been associated with arachnoid cysts include psychosis, paranoid delusions, alexithymia, attention deficit, hyperactivity, feeling of guilt, hallucinations, anxiety, depression, insomnia, irritability, cognitive disturbances, neurodevelopmental disturbances, and personality disorders [5, 6, 7, 8]. Usually, some of the aforementioned symptoms are reversible, and a significant improvement is observed after surgical decompression [9, 10]. Other studies have pointed out that arachnoid cysts impair not only basic cognitive ability but also executive functions [11]. Researchers have demonstrated that the cognitive impairment of patients seems to normalize after surgical decompression of the cyst, which further supports an etiological relationship [12].

A prospective cohort study conducted by Gjerde et al. [13] brings to light some reports of severe psychiatric symptoms and disorders in patients with arachnoid cysts, suggesting a possible etiological relationship between arachnoid cysts and mood disorders. Gjerde and his team have concluded that patients with arachnoid cysts have higher levels of anxiety and depression in comparison with the general population and that the symptoms normalize after decompressive surgery. Out of the 20 patients who underwent a craniotomy with cyst fenestration, 19 of them experienced clinical improvement in their depression and anxiety scores, with 16 of the patients achieving complete symptom relief. Furthermore, the researchers described a hemispheric asymmetry: patients with arachnoid cysts located in the right temporal lobe presented with higher anxiety and depression scores than patients with left temporal cysts. In contrast, psychosis was mostly associated with cysts in the left hemisphere. Temporal lobe disturbances are not confined to depression and anxiety; there are cases in the literature describing a relationship between left temporal lobe impairments and schizophrenia [14, 15, 16]. Cases have also been documented in which neurodevelopmental disorders have been related to temporal arachnoid cysts [10].

In our case, the patient presented with a right‐sided cyst and high depression scores; this is consistent with Gjerde's findings of hemispheric asymmetry. Furthermore, we encountered the same asymmetry in a previous case we published [9]. The patient who presented with delusions, anxiety attacks, and depression also had a right‐sided arachnoid cyst of possibly congenital origin. Regarding imaging techniques, a cohort study conducted by LeBaron et al. [17] does not support the practice of ordering structural imaging tests in the young adult psychiatric population. According to their findings, there was no significant improvement in patient management with the use of imaging techniques. On the other hand, the literature supports the importance of requesting them in cases of uncommon symptomatology that do not respond to pharmacological treatment [18]. In our case, we requested imaging tests due to our patient's unconventional symptom presentation and our hospital's imaging protocol for new patients. In the DTI MRI, we observed alterations from normal findings that could be related to the behavioral and temperamental changes and the depressive symptoms of the patient. It is worth highlighting a marked asymmetry with a higher MD value on the right side (lesion side), indicating less diffusion restriction on the side of the lesion. Likewise, the right side of most of the white matter tracts had higher FA values than the left side, except in the internal capsule (anterior and posterior arm), the cerebral and cerebellar peduncle, and the superior fronto‐occipital fasciculus. The major FA differences were in the cingulum (hippocampus), tapetum, uncinate fasciculus, and fornix. According to the literature [19], the cingulum (hippocampus and cingulate gyrus), the sagittal striatum, and the corticospinal tract should have an asymmetry to the left, but in our case, there is an asymmetry to the right. This asymmetry could be due to a compensatory reaction, but we do not have enough data to make a strong claim.

As for surgical treatment, there is still a lack of indication for surgery in this patient population presenting with psychiatric symptoms. The usual indications of arachnoid cyst surgery include intracranial hypertension, hydrocephalus or focal symptoms, intracranial hemorrhage, and those that show progressive growth in imaging tests. A watchful waiting attitude should be considered in small‐volume cysts, especially in asymptomatic cysts that were incidental findings. Fortunately, we are seeing an increase in cases where surgical intervention leads to partial or complete symptomatic relief. The aforementioned study by Gjerde et al. [13] is the first prospective study examining the pre‐ and postoperative anxiety and depression scores in patients with arachnoid cysts. They did not establish a causal relationship between intracranial arachnoid cysts and mood disorders but suggested an association. In the case we previously published [9], we achieved significant symptom resolution in a patient who was resistant to standard pharmacological treatment. In our current case, after not achieving any improvement with medical treatment, we contacted the neurosurgical department for a surgical evaluation, but they refused to operate and instead proposed an active follow‐up.

Taking into account our own clinical experience with previous cases of arachnoid brain cysts and the exclusive presence of psychological symptoms (without neurological symptoms) and our review of the current scientific literature, we cannot rule out that the patient's personality has been compromised in its normal development by alterations in brain neurodevelopment, and we consider it important to evaluate the possibility of surgical intervention in this patient population.

## Author Contributions

**Ruth Arias‐Hidalgo:** conceptualization, project administration, writing – original draft. **Felipe Ortuño:** project administration, supervision, writing – review and editing. **Sergio Manuel Solís‐Barquero:** conceptualization. **Maria Fernández‐Seara:** conceptualization. **Reyes García de Eulate:** conceptualization. **Aziz Yarkin:** writing – review and editing.

## Funding

The authors have nothing to report.

## Consent

Written informed consent was obtained from the individual(s) for the publication of any potentially identifiable images or data included in this article.

## Conflicts of Interest

The authors declare no conflicts of interest.

## Data Availability

The data that support the findings of this study are available from the corresponding author upon request.
